# Management of Self-Injurious Behavior, Aggression, and Psychogenic Non-Epileptic Seizures in Patients With Tuberous Sclerosis: A Case Report With a Review of Literature

**DOI:** 10.7759/cureus.11715

**Published:** 2020-11-26

**Authors:** Juan Fernando Ortiz, Samir Ruxmohan, Jonathan Gomez, Hector A Lalama, Amanda Taylor, Derrick Wayne Smith

**Affiliations:** 1 Neurology, California Institute of Behavioral Neurosciences & Psychology, Fairfield, USA; 2 Neurology, Larkin Community Hospital, Miami, USA; 3 Anesthesiology, Larkin Community Hospital, Miami, USA; 4 Psychiatry, American University of the Caribbean, Pembroke Pines, USA

**Keywords:** tuberous sclerosis, self injurios behavior

## Abstract

Tuberous sclerosis complex (TSC) is a neurocutaneous disorder that affects multiple systems. TSC encompasses neurobehavioral abnormalities that are considered less sensitive and specific to the disease. Autism spectrum disorder, attention deficit disorder, anxiety, mood disorders, and self-injurious behavior (SIB) are neurobehavioral manifestations associated with tuberous sclerosis. Among them, SIB is poorly described and studied. We present a case report and a brief review of the literature, which offers us insight into the pathological mechanism that explains associated SIB in TSC patients and provides a possible multidisciplinary approach to handle this complicated association. The case details a 21-year-old female with tuberous sclerosis who went to the emergency department and then transfer to the psychiatric floor due to aggressive behavior and SIB. The patient had a history of infantile spasm in childhood and generalized tonic-clonic seizures (GTCS); the last episode was four years ago at the age of 17. During the hospital admission, the patient developed an apparent tonic-clonic seizure. Nevertheless, the electroencephalogram (EEG) shows no epileptiform pattern and because of the clinical presentation, it was concluded she had psychogenic nonepileptic seizures (PNES). The patient's CT scan showed a stable appearance of multiple calcified subependymal nodules and left frontal hypodensity. Mini-mental examination (MMSE) revealed mild cognitive impairment. Patients with TSC/SIB have higher frequencies of mental retardation, TSC2 mutations, history of infantile spasms, spike focus in the left frontal lobe. Also, TSC/SIB patients have a higher frequency of tubers in quadrants other than the left posterior neuroanatomical region in left occipital, parietal, and posterior temporal lobes. Our patient had four out of five of the risks factor for developing TSC/SIB. Almost all patients with tuberous sclerosis are expected to develop seizures. Nevertheless, our patient was seizure-free for two years and managed prophylactically with antiepileptic medication. PNES can also occur in patients with tuberous sclerosis. It is essential to be attentive to differentiate PNES from actual seizures due to their history of the high frequency of seizures in TSC. Given the multiple systems involved in the symptomatology of TSC, including the SIB and neurological concerns, multidisciplinary treatment strategies must be implemented. Treatment of TSC with SIB should include antiepileptic drugs covering seizures and managing the SIB's mood component. A neuroleptic could be added for patients who are difficult to manage.

## Introduction

Tuberous sclerosis complex (TSC) is a neurocutaneous disorder that results in genetic defects in the hamartin and tuberin gene on chromosome 9 and 16, respectively. TSC affects multiple systems and presents with skin, cardiovascular, pulmonary, renal, neurological, and psychiatric abnormalities [1]. Common psychiatric conditions associated with TSC are autism spectrum disorder (ASD), attention deficit hyperactive disorder (ADHD), anxiety, mood disorders, and self-injurious behavior (SIB). Contrary to other disease manifestations, the behavioral abnormalities in TSC are less frequent and specific [2].

The neurobehavior abnormalities are less specific for TSC. Nevertheless, they have been described and studied. Self-injurious behavior is least been less described than other TSC's neurobehavior abnormalities, so the clinical picture of SIB associated with TSC remains mostly unknown. SIB refers to any behavior that harms one’s own body, which makes patients prompt to have suicide attempts [3]. We present a patient with TSC and SIB, pseudoseizures, and multiple suicide attempts. We conducted a brief literature review of the SIB mechanism in patients with TSC and proposed management options for this specific aspect of the condition, which is often overlooked due to the varying presentation of TSC.

## Case presentation

Initial presentation

A 21-one-year-old African American female with a past medical history of TSC was admitted to the psychiatric floor due to aggressive behavior and SIB at her home. The patient had multiple cuts on her body. The mother reported she was physically and verbally abusive towards her. Her home medication regimen included haloperidol, quetiapine, topiramate, and lorazepam. The neurology team came after the patient displayed seizure-like activity in the hospital. She suddenly felt weak and dizzy during a group therapy session. She began to have involuntary shaking of her head from side to side, and then the shakiness progressed to both the upper and lower limbs on the right side of her body. There was no loss of consciousness, auditory or visual auras, tongue biting, urinary incontinence, or fecal incontinence, but there was frothing at the mouth. The episode was terminated with one dose of 2 mg lorazepam.

Past medical history 

The patient was initially diagnosed with TSC when she was six years old due to skin manifestation (angiofibromas, ash leaf spots, and infantile spasms). At 12 years of age, she and her family moved to Florida, and was diagnosed with an oppositional defiant disorder. She was subsequently hospitalized at age of 13 after “striking mom in the temples with her fists.” She was reported to be “aggressive, agitated, anxious, and yelling in the middle of the street at the age of 13. The medication regimen at that time were quetiapine, valproic acid, clonidine, and sirolimus. At the age of 14, she is diagnosed with an angiolipoma on a CT scan. The patient has a history of two suicide attempts by cutting her wrist, the first one at the age of 16, and the second one when she had 19 years old. The dose of quetiapine was increased after the first autolytic attempt. Besides the infantile spasm, the patient has a history of tonic-clonic seizures with unknown duration. The last episode was at the age of 17. The patient has been seizure-free for two years. Table 1 details the patient medical history in chronological order.

**Table 1:** Past medical history of the patient in chronological order TSC: tuberous sclerosis complex

	Condition	Age
1	Formally diagnosed with TSC due to infantile spasm, ash leaf spots, and hamartoma in the iris	6
2	Oppositional defiant disorder	12
3	Aggressive behaviors, self-injurious behavior age	13
4	Angiolipoma	14
5	First autolytic attempt, second autolytic attempt	16 and 19
6	Tonic-clonic seizures, last episode at the age of 17	Unknown

Hospital admission 

**Figure 1:**
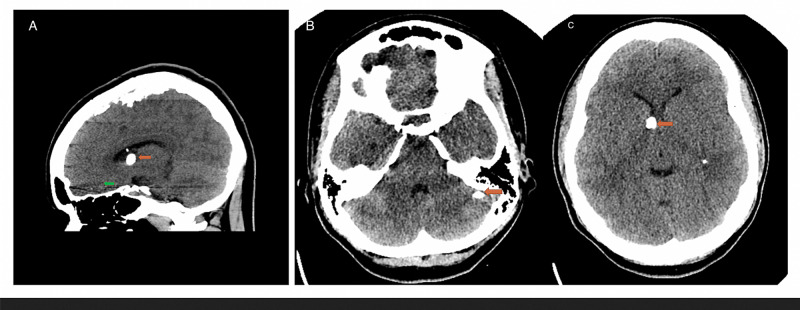
(A) CT, sagittal view, showed calcification in the temporal lobe under the knee of the corpus callosum (red arrow), and also shows frontal lobe hypodensity (green arrow). (B) CT, transversal view, shows calcification in the right cerebellar hemisphere (red arrow). (C) Transversal view shows a calcification at the level of the posterior horn of the left ventricle (red arrow)

During her hospital admission, she was followed by psychiatry and neurology. A CT scan of the brain showed multiple calcified subependymal nodules and left frontal hypodensity. The electroencephalogram (EEG) showed an abnormal slow-wave pattern. The EEG pattern thought to be because of her medication (valproic acid). On the EEG there were no spikes that suggest a seizure-like activity. Laboratory tests showed high prolactin (PRL) and creatine kinase (CK). We proceed to do a mini-mental examination (MMSE); the result was 22, suggesting mild cognitive impairment. Table 2 showed laboratory findings from the first four days of admission, and Figure 1 showed the CT scan with contrast of the patient. Imaging showed a stable appearance of multiple calcified subependymal nodules and left frontal hypodensity.

**Table 2:** Laboratory findings WBC: white blood cell count; AST: aspartate aminotransferase; ALT: alanine aminotransferase; CK: creatinine kinase

Work up	Day 1 (Admission)	Day 2	Day 3	Day 4	
WBC	11.2	8.86	8.3	7.9	4.80-10.8 × 10^3^/μL
Neutrophils	63.3%	53.3%	43.5	44.8	44-70%
Platelets	325	325	330	322	150-400 × 10^9^/L
Hemoglobin	11.5	11.3	11.2	11.6	12-16 g/dL
NA	137	135	137	136	136-145 mmol/L
K	4.6	3.7	4.1	4.0	3.5-5.1 mmol/L
Cl	107	105	106	106	98.107 mmol/L
Ca	9.1	9.1	8.7	8.7	8.6-10.3 mmol/L
AST/ALT	28/32	25/31	22/30	24/27	8-40 U/L
CO_2_	22	21	22	24	22-29 mmol/L
BUN	16	13	19	13	7-18 mg/dL
Creatinine	1.1	0.8	0.76	0.81	0.7-1.2 mg/dL
Glucose	96	90	96	90	70-100 mg/dL
Prolactin	40.92				0-20 ng/dL
CK	190				10/70 U/L
Topamax Level	8.2	7.6		7.6	5-20 μg/mL
Free Valproic acid		9.0		9.0	5-25 μg/mL

 

Diagnosis, treatment and follow up

The patient did not have a loss of consciousness, tongue bite, postictal confusion, or sphincter loss of control. The patient's head moves from side to side, the limbs' movement out of phase, and a normal EEG pattern makes us diagnose the patient with a pseudo seizure. The SIB diagnosis was evident because of the aggression and harm to the patient's own body.

The patient received optimal medical treatment and remained stable. After seven days of hospitalization, the patient was discharged with valproic acid, topiramate, and quetiapine. We continued the home medications and increased the dose of valproic acid. The idea of using valproic acid and topiramate is to cover the broad spectrum of seizures in the patient. While the combination of valproic acid and quetiapine for the SIB and the aggression. During the follow-up phone call one week after discharging the patient, there were no reported seizures, SIB, or any other complaints.

## Discussion

Psychogenic nonepileptic seizures in TSC

Psychogenic nonepileptic seizures (PNES) or pseudoseizures are functional disorders that could be seen as a conversion or dissociative disorder [4]. Because 79-90% of patients with TSC have epilepsy, is expected to miss PNES [5]. So, when psychogenic nonepileptic seizures are seen it expected to miss them.

The patient had an initial elevation of PRL and CK levels suggesting an actual seizure. PRL and CK are usually elevated after convulsions. However, in actual seizures, the elevations of PRL are seen about 20 minutes after seizures, and levels decrease after 60 minutes on average, but it significantly diminishes in the next 24 hours [6]. Postictal prolactin elevations are more frequent in women than men. Our patient's prolactin levels were 41 μg/dL, which are inferior to the average of 700 μg/dL in actual seizures [6]. The patient was using antipsychotics as part of her treatment. So, PRL elevation is likely due to her antipsychotics used [7]. While the sensitivity and specificity of PRL in seizures are unclear, it is an excellent diagnostic clue to support a recent seizure. PRL and CK levels are not elevated in patient with PNES. A study by Javali et al. found that 75% of patients with generalized tonic-clonic seizures (GTCS) had an increased level of PRL with a mean of 1379 U/L. While none of the patients with PNES have increased levels of PRL and had a mean of CK of 58 U/L [8]. 

Management of psychogenic nonepileptic seizures

Because psychogenic nonepileptic seizures is a functional disorder, it is better to be treated with cognitive behavior therapy (CBT). A study by Goldstein et al. showed that the patients treated with CBT had less frequency of "seizures" per month as compared with the control group (2.0 vs. 6.8) with an odds ratio (OR) 3; 95% CI 0.85-11.5) is compared to patients without CBT [9]. 

TSC/SIB proposed mechanism 

According to Eden et al., the rate of aggression and SIB in TSC is 50% and 27%, respectively [10]. Localizing the anatomic region is a starting point for improving the outcome of SIB in TSC patients. According to Gibson et al., there is a correlation between the low volume of the globus pallidus and the caudate in children with TSC [11]. Regarding critical associations, patients with TSC/SIB have higher frequencies of mental retardation, TSC2 mutations, history of infantile spasms, and spike focus in the left frontal lobe, a region prone to abnormal behavior when damaged [12]. Additionally, patients with SIB-associated TSC have a higher frequency of tubers in quadrants other than the left posterior neuroanatomical region in the left occipital, parietal, and posterior temporal lobes [11]. Wilde et al. found that the odds of having aggression (OR=2.6) and self-injury (OR=1.9) were higher in TSC/SIB's patients with intellectual disability. Another critical finding regarding correlations was that patients with autistic spectrum disorder (ASD) have an increased risk of developing TSC/SIB [13]. 

Our patient has various risk factors of TSC/SIB. Tubers in quadrant other than the left posterior neuroanatomical region, MMSE of 22 (mild cognitive impairment), history of infantile spasm, making the TSC/SIB almost predictable and expected. Regarding aggression, it was seen that low mood, high levels of activity, intellectual disability, and symptoms of ASD make the patients with TSC more prone to be aggressive [10]

TSC/SIB and aggression management**

TSC patients with intellectual disability, ASD, ADHD, or spike focus in the left frontal lobe should be screened for SIB [12,13]. Moreover, with higher behavioral complaints directly correlated with higher seizure frequency rates, it is wise to endorse a multidisciplinary approach to managing TSC and improving SIB [14]. Given the combined concerns of epilepsy, mood disruptions, and maladaptive conduct like aggression and self-injurious behavior, ideal treatment plans should adequately improve the symptoms and quality of life for TSC patients with minimal burden. In our research, lamotrigine and valproic acid are excellent options in managing both the seizure and behavioral components of TSC. Lamotrigine is a reasonably effective antiepileptic drug utilized with TSC patients in the management of seizures. A study found that 57 TSC patients utilizing lamotrigine that 42% became seizure-free, and 37% experienced an >50% reduction in seizure frequency [14]. Interestingly, there was also a 32% improvement in patient behaviors and alertness of daily activities, which argues that lamotrigine has treatment impact beyond solely seizure management. 

Valproic acid is an antiepileptic with a vast spectrum of action. It can be used in many clinical indications, versus all other antiepileptic drugs in treating adults and children [15]. In a study that included 71 TSC patients treated for epileptic seizures between 1988 to 2014, valproic acid was the most frequently used anticonvulsant at 85% of the participants [16]. Additionally, valproate is the drug more initially prescribed as an anticonvulsant in TSC patients. According to Overwater et al., the drug was prescribed 45% of the time [16]. It was reported that 52% of patients became seizure-free after initiating valproic acid as the first antiepileptic drug. In considering TSC associated behavioral concerns like depression, hyperactivity, and aggression, valproic acid's broad clinical implications can prove beneficial. With these clinical benefits in mind, valproic acid has become the most utilized mood stabilizer in patients with schizophrenia over the past decade despite this indication being off-label [17]. 

## Conclusions

Almost all patients with tuberous sclerosis are expected to develop seizures. Nevertheless, our patient was seizure-free for two years. PNES can also occur in patients with tuberous sclerosis. It is essential to differentiate PNES from actual seizures and be biased because of the high frequency of patients with TSC having seizures. The patient had an elevation of PRL value. This elevation could have been caused by neuroleptic medication. Like we mentioned before, CK levels can also be increased in PNES. The EEG pattern showed a negative pattern of seizures. Patients with TSC/SIB have higher frequencies of mental retardation, TSC2 mutations, history of infantile spasms, spike focus in the left frontal lobe. Also, TSC/SIB patients have a higher frequency of tubers in quadrants other than the left posterior neuroanatomical region in the left occipital, parietal, and posterior temporal lobes. Our patient does not have mental retardation but had mild cognitive impairment. The gene mutations were not measured. The patient had a history of infantile spasm, had tubers in quadrants other than the left posterior neuroanatomical region in the left occipital, parietal, and posterior temporal lobes. To summarize, our patient had four of five risk factors for SIB that we find in our research related to TSC.

TSC/SIB affects multiple systems and requires a multidisciplinary approach. The drugs like valproic acid or lamotrigine that preferentially cover SIB and TSC at the same time are preferred. These drugs prevent seizures and also regulate mood in patients with TSC. 
